# Supplementary material for: Combining stem cell rejuvenation and senescence targeting to synergistically extend lifespan
Source: Aging (Albany NY). 2022 Oct 25;14(20):8270–91. doi: 10.18632/aging.204347 (PMC9648810; doi:10.18632/aging.204347)
Supplement: Supplementary Figures [file aging-14-204347-s001.pdf]

SUPPLEMENTARY FIGURES

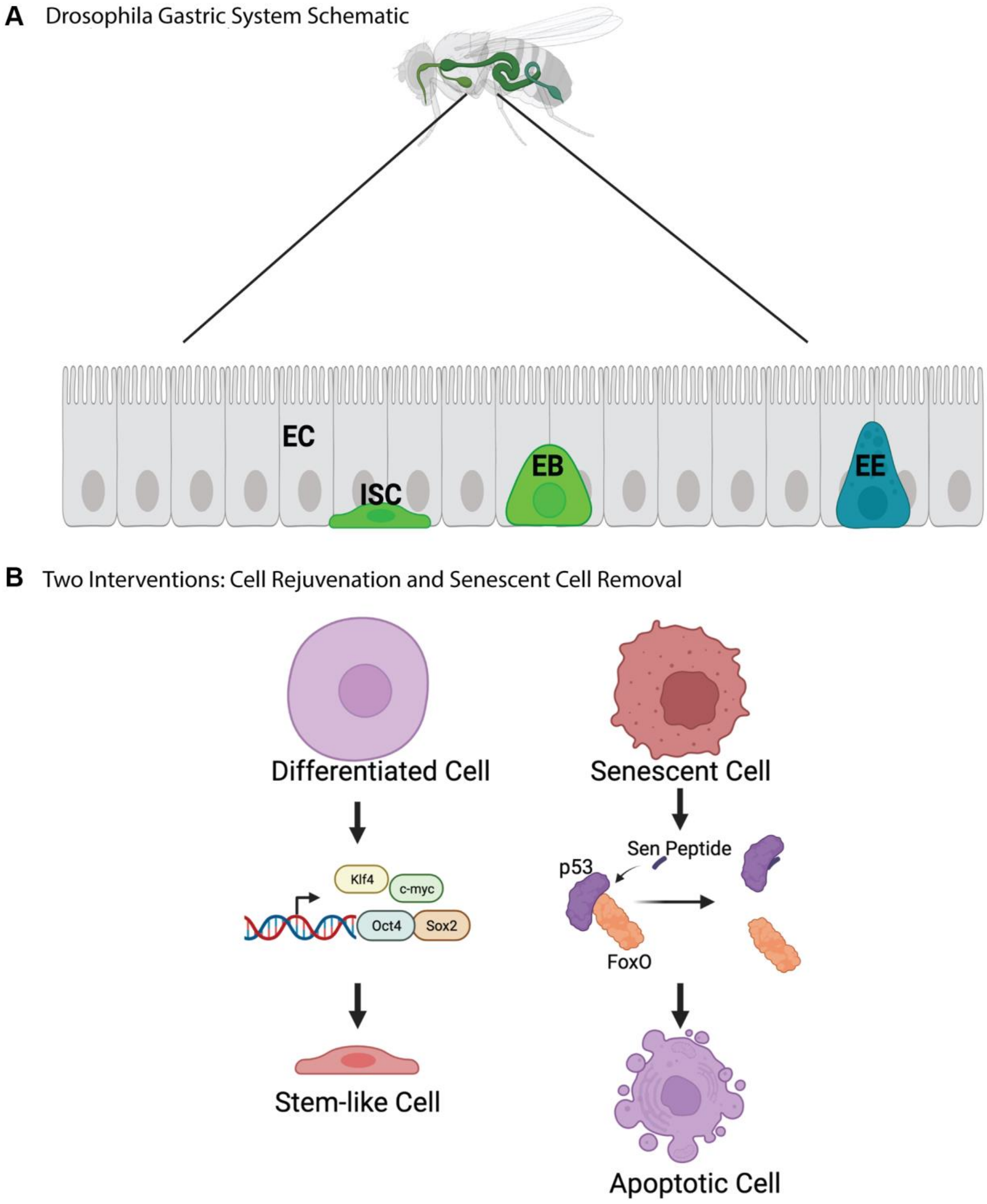

Supplementary Figure 1. (**A**) Schematic of the Drosophila digestive system comprising enterocytes (ECs), enteroendocrine (EEs), enteroblasts (EBs) and intestinal stem cells (ISCs). ISCs are located externally, away from the lumen. They divide symmetrically to make more ISCs or asymmetrically to form EBs or transit amplifying cells that further differentiate into ECs and EEs. (**B**) Model for the two treatments, rejuvenating differentiated cells to more stem cell like cells through Yamanaka factor expression, and activating apoptosis of senescent cells through peptide-based interference in FoxO-p53 binding.

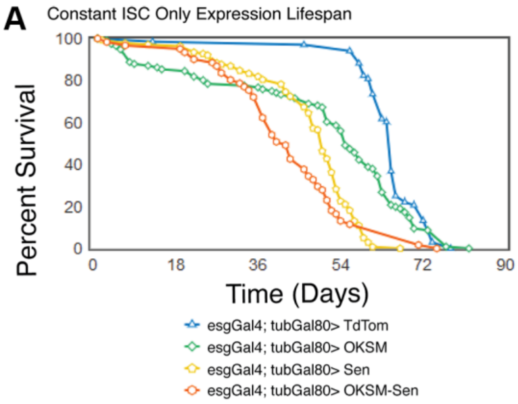

Condition	Number of samples	Mean lifespan		Max Lifespan (90th percentile)	
		Age in days	p-value versus TdTom (Log-rank test)	Age in days	p-value versus TdTom (Fisher's exact test)
esgGAL4; UAS-GFP; TubGal80 TdTom	68	64.41	-	74.00	-
esgGAL4; UAS-GFP; TubGal80 OKSM	116	49.66	0.003 (**)	70.00	0.215
esgGAL4; UAS-GFP; TubGal80 Sen	138	46.60	0.000 (**)	59.00	0.000 (**)
esgGAL4; UAS-GFP; TubGal80 OKSM-Sen	61	42.57	0.000 (**)	71.00	0.019 (*)

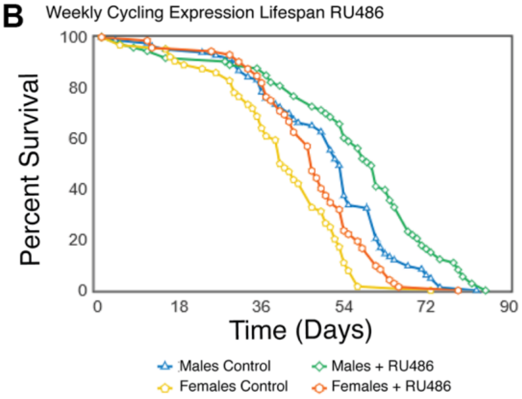

Gender	Condition	Number of samples	Mean lifespan		Max Lifespan (90th percentile)	
			Age in days	p-value, Ethanol versus RU486 (Log-rank test)	Age in days	p-value, Ethanol versus RU486 (Fisher's exact test)
Actin-Switch GFP; UAS-OKSM Males	Ethanol Control	83	50.04	0.006 (**)	68.00	0.054
	RU486	73	55.86		79.00	
Actin-Switch GFP; UAS-OKSM Females	Ethanol Control	64	40.41	0.018 (*)	55.00	0.003 (**)
	RU486	72	46.68		61.00	

Supplementary Figure 2. Lifespan study of constitutive OKSM, Sen and OKSM-Sen expression in fly guts. (**A**) Expression of OKSM, Sen and OKSM-Sen in *escargot* expressing cells of guts of adult female flies resulted in significantly reduced lifespan as compared to control flies (esgGFP TdTom). (**B**) Induced expression of OKSM, Sen and OKSM-Sen (post-eclosion) in separated male and female flies using drug induced expression resulted in significantly increased lifespans for both male and female flies. A *P*-value of 0 reflects $P < 1.0 * 10^{-10}$.

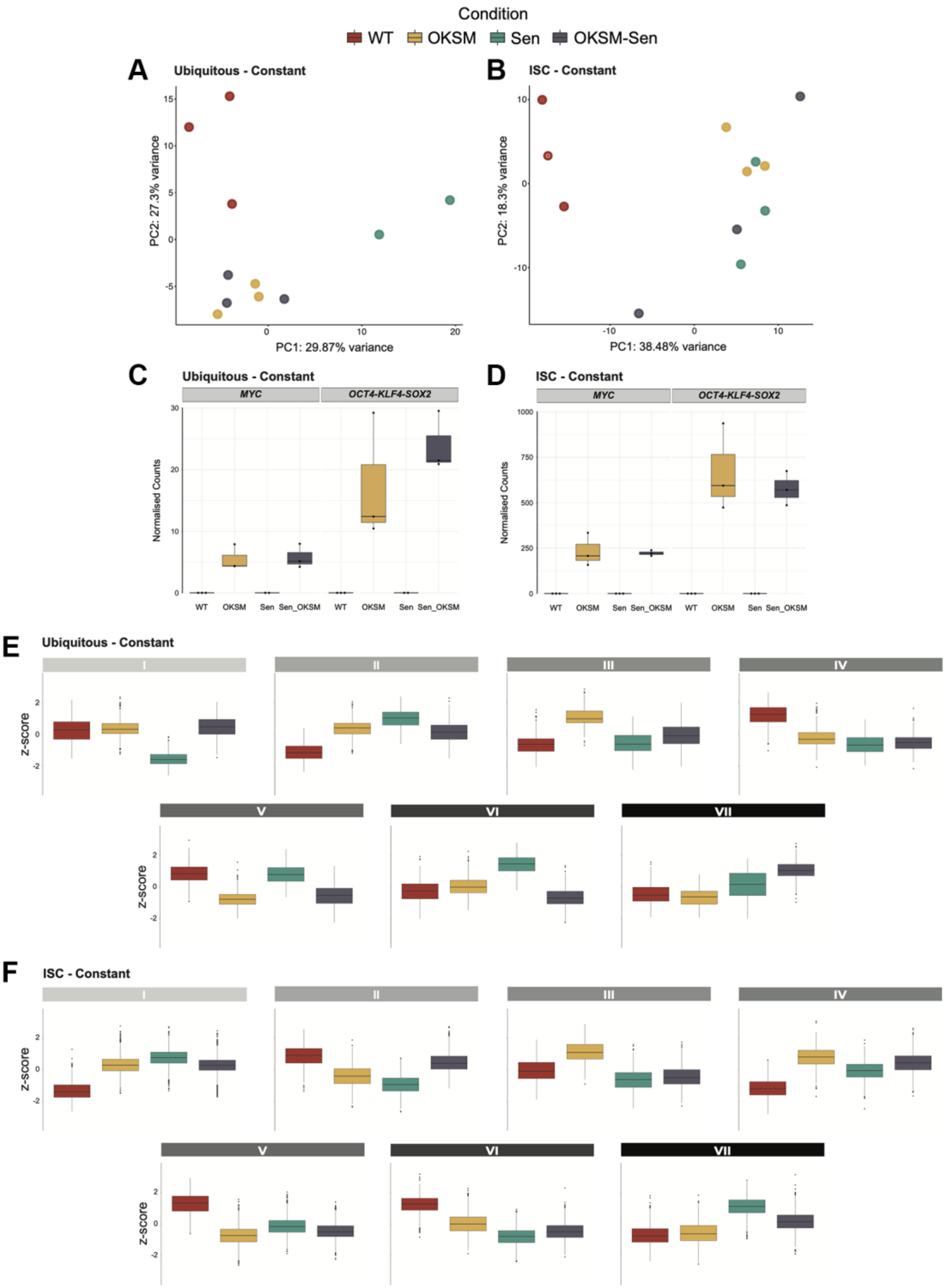

Supplementary Figure 3. Exploratory Data Analysis (EDA) of gene expression changes in the *Drosophila* gut in Sen, OKSM and OKSM-Sen treatment. (**A**) PCA plots of gene expression in ubiquitous expression experiments with armGal4; tubGal80^{ts} > UAS-TdTomato (WT), armGal4; tubGal80^{ts} > UAS-OKSM (OKSM), armGal4; tubGal80^{ts} > UAS-Sen (Sen) and armGal4; tubGal80^{ts} > UAS-Sen; UAS-OKSM (OKSM-Sen). (**B**) PCA plots of gene expression in stem cell only expression experiments with esgGal4; tubGal80^{ts} > UAS-TdTomato (WT), esgGal4; tubGal80^{ts} > UAS-OKSM (OKSM), esgGal4; tubGal80^{ts} > UAS-Sen (Sen) and esgGal4; tubGal80^{ts} > UAS-Sen; UAS-OKSM (OKSM-Sen). Quantification of the expression of the OKSIM (*OCT4-KLF4-SOX2*-IRES-*MYC*) construct in (**C** The ubiquitous expression model and **D**) in the stem cell only expression model (**E**) Z-scores boxplots of ubiquitous expression experiments with armGal4 for each of the seven clusters (**F**) Z-scores boxplots of ISC-restricted expression model with esgGal4 for each of the seven clusters.

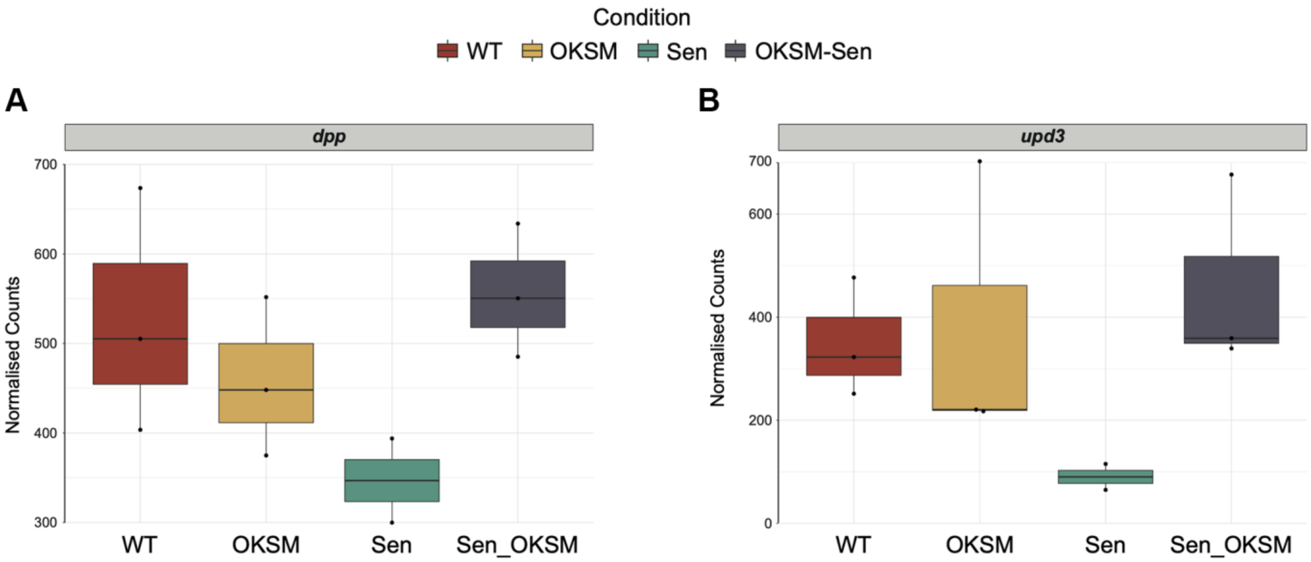

Supplementary Figure 4. Expression of (**A**) *dpp* and (**B**) *upd3* in the ubiquitous constant expression model.
