# Supplementary material for: Combining stem cell rejuvenation and senescence targeting to synergistically extend lifespan
Source: Aging (Albany NY). 2022 Oct 25;14(20):8270–91. doi: 10.18632/aging.204347 (PMC9648810; doi:10.18632/aging.204347)
Supplement: Supplementary Table 3 [file aging-14-204347-s004.pdf]

SUPPLEMENTARY TABLE

Supplementary Table 3. Fitting parameters and statistics from Gompertz–Makeham mortality and survival analysis.

Induction: Continuous	MRDT			A0				
	low95	med	high95	low95	med	high95	RSE	df
Control	11.3	11.9	12.6	81.2	100	118.9	0.026	45
OKSM	10.4	11.2	12.1	511.1	595.4	679.6	0.033	48
Senolytic	11.3	12.1	13	254.7	303.5	352.4	0.032	48
Synergy	5.9	6.5	7.2	34.5	61.4	88.2	0.042	35
Additive	9.9	11.4	13.2	1301.7	1807.1	2394.9	NA	NA
**Induction: 24 h**	**MRDT**			**A0**				
	**low95**	**med**	**high95**	**low95**	**med**	**high95**	**RSE**	**df**
Control	20.5	22.4	24.6	76.2	100	123.9	0.04568	52
OKSM	17.6	18.3	19	46.8	54	61.3	0.0199	54
Senolytic	8.5	9	9.5	0.2	0.4	0.6	0.0251	43
Synergy	11.1	12	13	0.7	1.6	2.5	0.0339	44
Additive	6.7	7.4	8.1	0.1	0.1	0.3	NA	NA
**Induction: 12 h**	**MRDT**			**A0**				
	**low95**	**med**	**high95**	**low95**	**med**	**high95**	**RSE**	**df**
Control	21.6	22.7	24	85.9	100	114.1	0.0246	50
OKSM	18.6	19.6	20.7	23.3	29.9	36.4	0.0336	64
Senolytic	7.5	7.9	8.3	0.04	0.1	0.1	0.0214	35
Synergy	7.7	8.2	8.9	0.002	0.03	0.06	0.0291	45
Additive	6.1	6.8	7.6	0.009	0.02	0.047	NA	NA

Best fit value and 95% confidence interval are listed for each parameter. Mortality rate doubling time is given in days and rounded to one decimal place. Initial mortality parameter A is normalized to that of the relevant control cohort (WT flies subjected to the same induction condition). All A parameter values are expressed in percent of this control.
